# Mitochondria as a therapeutic: a potential new frontier in driving the shift from tissue repair to regeneration

**DOI:** 10.1093/rb/rbad070

**Published:** 2023-08-12

**Authors:** Evan N Main, Thaiz M Cruz, Gary L Bowlin

**Affiliations:** Department of Biomedical Engineering, University of Memphis, 330 Engineering Technology Building, Memphis, TN 38152, USA; Department of Biomedical Engineering, University of Memphis, 330 Engineering Technology Building, Memphis, TN 38152, USA; Department of Biomedical Engineering, University of Memphis, 330 Engineering Technology Building, Memphis, TN 38152, USA

**Keywords:** mitochondrial transplantation, host–biomaterial integration, biomaterials, wound healing, fibrosis

## Abstract

Fibrosis, or scar tissue development, is associated with numerous pathologies and is often considered a worst-case scenario in terms of wound healing or the implantation of a biomaterial. All that remains is a disorganized, densely packed and poorly vascularized bundle of connective tissue, which was once functional tissue. This creates a significant obstacle to the restoration of tissue function or integration with any biomaterial. Therefore, it is of paramount importance in tissue engineering and regenerative medicine to emphasize regeneration, the successful recovery of native tissue function, as opposed to repair, the replacement of the native tissue (often with scar tissue). A technique dubbed ‘mitochondrial transplantation’ is a burgeoning field of research that shows promise in *in vitro*, *in vivo* and various clinical applications in preventing cell death, reducing inflammation, restoring cell metabolism and proper oxidative balance, among other reported benefits. However, there is currently a lack of research regarding the potential for mitochondrial therapies within tissue engineering and regenerative biomaterials. Thus, this review explores these promising findings and outlines the potential for mitochondrial transplantation-based therapies as a new frontier of scientific research with respect to driving regeneration in wound healing and host–biomaterial interactions, the current successes of mitochondrial transplantation that warrant this potential and the critical questions and remaining obstacles that remain in the field.

## Graphical abstract

**Figure:**
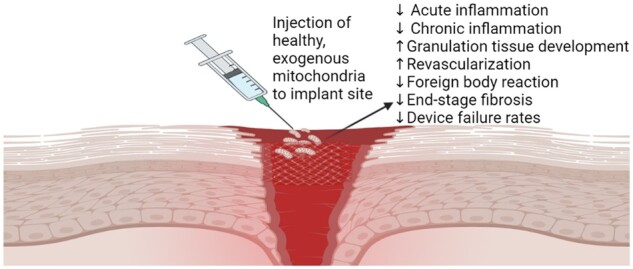

## Introduction to the host–biomaterial response and wound healing continuum

When an injury occurs, whether due to trauma or the implantation of a biomaterial, a cavalcade of intricate and interweaving mechanisms is set into motion, with the end goal being to restore homeostasis. This host response to injury is a series of interdependent processes wherein the success of one phase is determined by the successful resolution of the preceding phase. These phases, listed in Table 1, are intricate and depend on the proper microenvironment to be fully and well resolved [1, 2]. During each phase, key cell types predominate to accomplish highly specialized tasks. Overall, the entire host response occurs within 2–3 weeks and varies from tissue, organ and species (this review will focus solely on human tissues).

**Table 1.** Different phases in the host response to injury [1]

Injury
Blood–material interaction
Provisional matrix formation
Inflammation
Granulation tissue development
Foreign body reaction
Fibrosis

### Injury

Injury, the initiating event of the host response continuum, can result from the surgery necessary to implant a given biomaterial, from trauma, from the loss and sudden regain of blood flow to tissue (ischemia–reperfusion injury) or from a myriad of other causes. The response to initial injury is affected by several parameters: the extent of the disruption to blood flow, the loss of anchoring membrane structures and the apoptosis or necrosis of surrounding cells [3]. The challenges and demands of the wound/implant environment will depend on the nature, magnitude and context of the damage. However, in the intervening phases of the host response, underlying tissue engineering opportunities exist to help shift the fate of the environment from repair to regeneration.

### Blood–material interaction and provisional matrix formation

After the initial injury, the body aims to stop the bleeding and to recognize any foreign materials that may be present in the wound microenvironment. The blood–material interaction (BMI) phase plays a dual-sided role in terms of implanted biomaterials. In the context of wound healing, blood protein buildup is known as provisional matrix formation, as it provides structure and many of the materials necessary to close a wound. However, thrombosis can block the device–tissue interface in biomaterial implantation or potentially risk embolism. The prevention of thrombosis is a crucial parameter that has been recognized in biomedical engineering and biomaterial design for years, with the inclusion of heparin in vascular prosthetics being standard practice. Including a localized anticoagulant decreases the risk of embolism or device blockage but at the expense of allowing proper integration of the material by impeding provisional matrix formation. Thus, a delicate balance must be struck during BMI and provisional matrix formation. An environment that is too thrombogenic can create blockages in the wound area or surrounding vasculature and potentially risk embolism [4, 5]. However, the development of a fibrin-rich provisional matrix is critical for the cessation of bleeding and the infiltration of support cells [6]. A wound environment that does not have a sufficient provisional matrix will fail to provide structure and nutrients for the upcoming phases of healing [7]. Deposition of blood proteins onto a material also primes the immune response by activating the complement system, targeting foreign objects for phagocytosis or degradation from immune cells, thus leading to the subsequent phases of the host response [1, 8].

### Acute inflammation

Inflammation ensues as a normal and necessary response to tissue damage and the presence of foreign materials or pathogens. Initially, neutrophils act as the first responding immune cells, recognizing signs of damage, such as damage-associated molecular patterns (DAMPs), or molecules found on the surface of pathogens, such as pathogen-associated molecular patterns (PAMPs) [9, 10]. Recent developments in neutrophil biology indicate that these ‘first responder’ cells have a much more complex and varied range of behavior than merely seeking and destroying [11]. Upon BMI and provisional matrix formation, small clusters of ‘pioneer’ neutrophils interacting with the wound microenvironment will initiate cell signaling to recruit additional neutrophils to the area. Depending on the degree of injury or infection, the ensuing neutrophil swarm can be categorized as ‘transient’ or ‘persistent’ [12]. Transient and persistent neutrophil swarming patterns have been observed in a wide range of tissues and are found to be highly conserved between species, with comparable dynamics observed in zebrafish larvae and mouse tissues. For a comprehensive review of neutrophil swarming, the authors suggest ‘In the eye of the neutrophil swarm—navigation signals that bring neutrophils together in inflamed and infected tissues’ by Lämmermann [13]. Briefly, neutrophil swarming dynamics have been evaluated in murine injury-mediated inflammation of skin and ear tissues, liver vascular occlusion, transplant-induced ischemia–reperfusion damage and brain amyloid plaque development [14–18]. Neutrophil swarming has also been observed in murine pathogen-mediated inflammation involving *Staphylococcus aureus*, *Listeria monocytogenes*, *Escherichia coli* and other pathogens [16, 18–20]. For example, murine models of parasite-infected lymph nodes (by direct injection of *Toxoplasma gondii* into earflaps or oral ingestion) observed and characterized such neutrophil swarming patterns. Transient swarms were characterized as highly organized neutrophils with cell numbers between 10 and 150 quickly entering the site of lymph node infection and dissipating after 10–40 min. Persistent neutrophil swarming involved a disorganized swarm of over 300 neutrophils demonstrating sustained inflammatory attack and signaling for over 40 min within the lymph nodes [12].

### Chronic inflammation

There is a delicate balance during the acute inflammatory phase between a sufficient immune response to ward off pathogens and a dysregulated immune response that can cause further tissue damage. Rampant and unresolved acute inflammation will then dovetail into higher levels of chronic inflammation. Chronic inflammation, unlike acute inflammation, is primarily macrophage mediated. Upon further DAMP and PAMP signaling, macrophages will polarize to an M1 phenotype, sustaining a persistent pro-inflammatory microenvironment [21]. The prolonged inflammatory response of both neutrophils and macrophages leads to a positive feedback loop wherein the damage accrued by oxidative and enzymatic degradation of the wound environment then releases more DAMPs, further recruiting immune cells [22]. The previous processes are similar in both the context of wound healing and the host–biomaterial response. However, after the induction of chronic inflammation, these paths diverge. The hostile environment created by an overabundant immune response will impede support cell infiltration and prevent any remodeling, leading to premature wound closure by fibroblast differentiation to myofibroblasts and resultant fibrosis [23]. From a biomaterial implantation perspective, a hostile microenvironment with consistent immune cell recruitment and activation can accelerate the degradation of the biomaterial and wall off the tissue–device interface [24, 25]. The results of the development of chronic inflammation are discussed in both contexts in the proceeding case studies.

## Injury case study: post-myocardial infarction fibrosis

As previously mentioned, other forms of injury besides trauma and biomaterial implantation can lead to fibrosis. Ischemia–reperfusion damage can cause widespread cell death, trigger inflammation and lead to a reparative fibrotic response. For example, after myocardial infarction, cell death can reach up to 1 billion cardiomyocytes due to ischemia [26]. This large-scale necrosis of characteristically non-proliferative cardiomyocytes leads to a disruption of extracellular matrix (ECM) homeostasis, a shift in fibroblast phenotype to myofibroblasts and the initiation of an inflammatory response [27]. In healthy cardiac tissue, the ECM is kept in balance by the secretion of collagen (mainly types I and III) by fibroblasts and the degradation of ECM fibers by matrix metalloproteinases (MMPs) [28]. In the wake of myocardial infarction, this homeostasis is disrupted. For example, after ischemia, MMP9 is found to be upregulated, causing degradation of the surrounding ECM. At the same time, both fibroblast apoptosis and differentiation into myofibroblasts lead to lower collagen deposition and decreased organization of the secreted collagen. With lower levels of MMP9 within the damaged microenvironment, lessened fibrotic signaling and lower levels of end-stage ventricular fibrosis were found in an aged mice model [29].

Additionally, ischemia damages the microvasculature in the infarct area, releasing similar signals to the vascular damage seen in surgery or trauma and compromising cardiomyocyte and endothelial cells’ respiratory capabilities [30]. As previously mentioned, when the body cannot regenerate a tissue, it shifts to a reparative process. The widespread loss of cardiomyocytes changes the path of the wound microenvironment toward repair. It primes fibroblasts' differentiation into myofibroblasts to close the cellular void and seal it with disorganized collagen. This deposition of scar tissue, without the replacement of parenchymal tissue, restores local homeostasis but at the cost of normal heart function. Fibrosis in the infarct border zone causes an alteration in cardiac chamber compliance, as well as an increase in the stiffness of ventricular tissue.

Additionally, interstitial fibrosis and scar tissue buildup in the myocardium have been found to disrupt the conduction of electrical signaling. The initiation of an immune response also leads to further tissue damage and resultant fibrosis. These factors predispose the heart to further myocardial infarction and arrhythmia [27].

After myocardial infarction, the large-scale necrosis of cardiomyocytes and the surrounding microvasculature induces an immune response [27]. Initially, myocardial cell necrosis releases mitochondria-rich subcellular membrane components capable of activating complement cascade components C1, C2, C3 and C4 [31]. Research has indicated that mRNA and protein components for all elements of the classical complement pathway within the infarct area are upregulated after ischemia–reperfusion [32, 33]. Interestingly, leukocyte chemotaxis within postischemic lymph peaks within the first hour of reperfusion and attenuates within the following three hours, drawing parallels to neutrophil swarming dynamics [34].

A vicious cycle is initiated upon neutrophil swarming to areas of large-scale necrosis. Upon pro-inflammatory activation from the infarct area, neutrophils release NADPH oxidase-derived superoxide, which becomes hydrogen peroxide through dismutation. Additionally, myeloperoxidase (MPO) release catalyzes the formation of hypochlorous acid as another source of oxidative tissue damage [35]. Further tissue damage ensues due to degradative enzymes released from neutrophil granules, primarily the proteases elastase and cathepsin G [35]. Studies have revealed a complex interplay between oxidative damage and protease-mediated tissue damage. *In vitro* data involving the co-culture of hepatocytes with activated neutrophils have shown that cell damage is largely protease mediated, with little to no involvement of reactive oxygen species (ROS) [36, 37]. However, *in vivo* studies have demonstrated enhanced ROS production from activated neutrophils and resultant lipid peroxidation in parenchymal tissues under inflammatory conditions [38, 39].

Additionally, antioxidant treatments ameliorated inflammation-driven liver injury in several *in vivo* models, further suggesting the role of ROS in pathological inflammation [40, 41]. To reconcile these contradictions, a fundamental principle must be considered. *In vitro* experiments provide only a microcosm of a complex interplay of the entire physiology present *in vivo*. Anti-proteases in the blood plasma are found to be disrupted by oxidation. Thus, ROS generation via neutrophils creates a microenvironment wherein their proteases can have a much more drastic (and potentially deleterious) effect [42]. This cascade of additional tissue damage from the acute immune response then primes the area for a more extensive amount of requisite remodeling and paves the way for chronic inflammation. Upon neutrophil-mediated cell signaling and DAMP-mediated signaling, there is a heightened presence of phenotypically pro-inflammatory macrophages within the wound microenvironment, tilting the course of the wound-healing continuum further towards additional damage, a delay in remodeling and ultimately fibrosis [43, 44].

The resultant tissue damage from the initial ischemia/reperfusion event and the subsequent inflammation leads to fibrosis in the post-myocardial infarction heart, leading to arrhythmia, loss of cardiac output and risk of further cardiac arrests discussed earlier. Ameliorating the effects of myocardial infarction has been a primary goal in medicine for decades, and numerous techniques have been proposed with varying degrees of success. Recently, a proposed avenue of research with promising clinical evidence has been in the field of mitochondrial transplantation. This review aims to discuss the history that led up to the current use of mitochondrial transplantation as a therapeutic, critically analyze the reported benefits and the mechanisms by which they occur and suggest ways in which mitochondrial transplantation can be used in other avenues, including, and especially, tissue engineering and the promotion of regenerative biomaterials.

## Host–biomaterial response case study: vascular prosthetic implants

A well-known and unfortunate phenomenon within the field of biomaterials is the development of a fibrotic capsule surrounding the implant [45]. Various factors contribute to the fibrous encapsulation of a biomaterial, including surface chemistry, porosity, mechanical stresses and many other factors, some of which have yet to be elucidated [46]. A specific application that has been plagued with this issue for over half a century is the development of vascular prosthetic biomaterials, especially small-diameter (<6 mm in diameter) vascular prosthetics [47]. The gold standard biomaterial used for synthetic vascular prosthetic implants since 1969 is expanded polytetrafluoroethylene (ePTFE) [48]. While ePTFE vascular prosthetics pass all ISO10993 tests for biocompatibility, it is a well-recorded phenomenon that ∼3 weeks after implantation, the graft is surrounded by a dense, poorly vascularized, collagenous capsule of typically 50–150 µm in thickness [47, 49, 50]. ePTFE implant-induced fibrosis occurs due to the development of thrombus occlusion, sustained immune attack leading to chronic inflammation at the device–tissue interface, and the fusing of activated macrophages into foreign body giant cells [50–53].

Similarly to the post-myocardial infarction development of fibrosis, recent developments have shown that the initial neutrophil response to an implanted biomaterial can set the path for the proceeding stages of the host–biomaterial response [43]. The host–biomaterial continuum follows the same general phases discussed previously regarding wound healing, with a strong dependence on the successful resolution of each phase to achieve regeneration of the host–device interface [2]. The unsuccessful resolution of the host–biomaterial response shifts the body to repair the interface instead of integrating with the biomaterial, laying down a dense network of scar tissue to wall off the material [46]. In some biomaterial applications, this can render the biomaterial useless or significantly less effective, such as in the cases of glucose monitor implants [54]. However, in some applications, it can be a significant obstacle to the development of new technologies and can leave significant gaps in the ability to treat diseases.

The effects of a poor host–biomaterial response are even more deleterious in small-diameter vascular prosthetics for the following reasons. The end-stage fibrotic capsule has been demonstrated as being non-uniformly constrictive, causing flow disturbances within the vessel, causing increased thrombosis. The capsule is also nonelastic, causing a discrepancy between the mechanical properties of the vasculature and the device, potentially causing hyperplasic cell proliferation [47]. Additionally, the avascularity of the device due to the fibrous capsule will prevent complete healing and reendothelialization of the vessel [55]. Finally, the sustained oxidative and enzymatic degradation by neutrophils and macrophages due to chronic unresolved inflammation can directly lead to device failure and rupture. Small-diameter grafts are far more prone to failure from these mechanisms because even small occlusions, flow rate disturbances or mechanical mismatches can significantly affect the total flow through such a small diameter.

Because of the limited ability of vascular prosthetics to resolve acute immunity and prevent ensuing chronic inflammation, there are currently no small-diameter synthetic vascular prosthetics approved by regulatory agencies in use in the clinic today [47]. The lack of synthetic vascular prosthetics with inner diameters below 4 mm available on the market leaves an enormous unmet need to prevent the ∼1 million limbs amputated worldwide yearly [48].

Thus, not only in the field of wound healing but also in the fields of tissue engineering and biomaterial implantation, there is a tremendous need for techniques and therapeutics that can modulate the host response toward regeneration. Mitochondrial therapies have shown great promise in a multitude of different pathologies. Perhaps this promise could also be realized to accomplish more complete and effective wound healing and integration of biomaterials.

## Introduction to mitochondria

**Figure 1.**
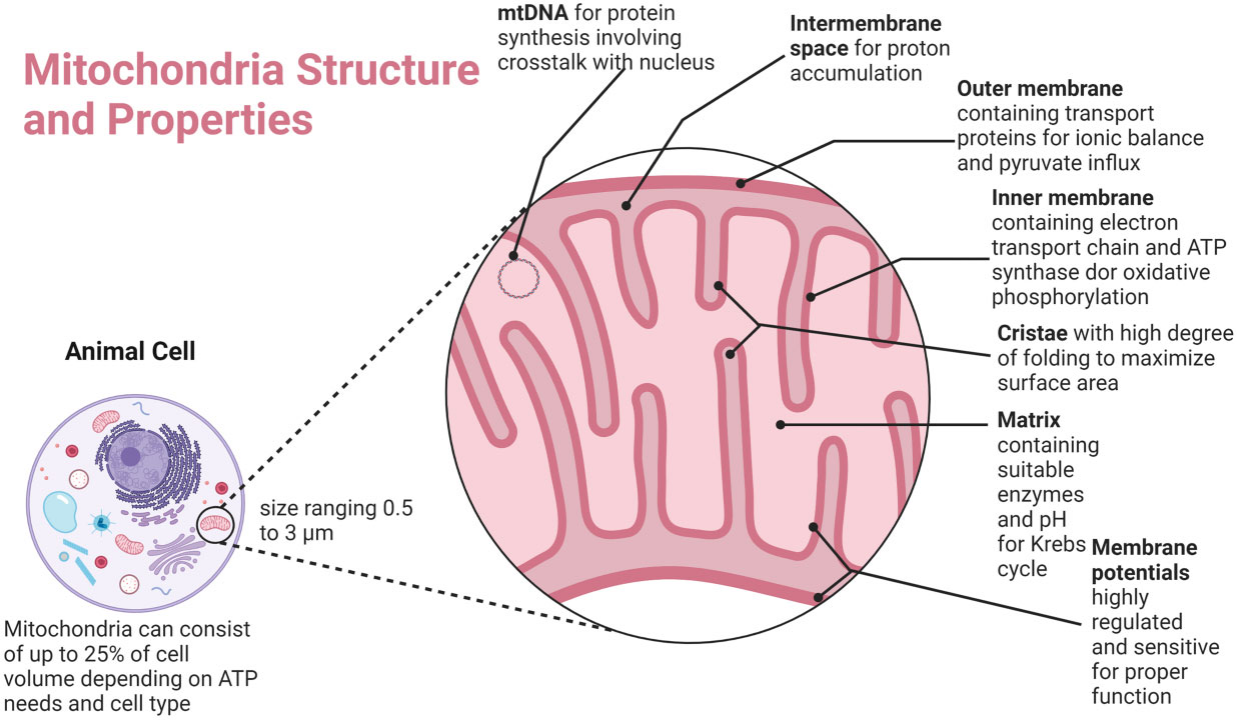
Overview of mitochondrial structure, characteristics and properties. Created with BioRender.com.

Mitochondria are dynamic organelles located within the cytoplasm of most eukaryotic cells and all mammalian cells except for mature red blood cells (Fig. 1) [56]. They produce most of the cell’s useful energy in the form of adenosine triphosphate (ATP) via the process of oxidative phosphorylation. As well as being the cell’s primary source of energy, mitochondria also play a crucial role in cell proliferation, production of metabolites, coordination of various metabolic pathways, heat regulation, assembly of iron–sulfur proteins, calcium homeostasis and apoptosis signaling via the release of cytochrome *c* [57–60]. A small number of oxidative molecules and free radicals are created through oxidative phosphorylation, called ROS. An overabundance of ROS can lead to oxidative stress, disrupting normal cellular function. When damaged, mitochondria produce less ATP and more ROS, leading to oxidative damage and a deficit of energy in the surrounding area. Mitochondrial dysfunction is a feature in many health concerns, such as cancer [61], heart failure [62], muscle atrophy [63], chronic inflammation [64] and aging [65]. Mitochondrial dysfunction also plays a vital role in traumatic injuries such as traumatic brain injury and ischemia due to myocardial infarction, permanently altering cellular function [66, 67]. It is abundantly clear that maintaining functional and viable mitochondria is critical in preserving and maintaining healthy tissue and avoiding inflammation. For a comprehensive review of mitochondrial structure, function and the effects of disease state versus healthy state on mitochondrial function and structure, the authors of this article suggest ‘Mitochondrial structure and bioenergetics in normal and disease conditions’ by Protasoni and Zeviani [68].

The crux of mitochondrial transplantation as a therapy and how mitochondria can be isolated, injected and taken in by cells in the body with no significant immune response lies in the evolutionary history of the mitochondrion. The current prevailing theory as to the origins of mitochondria suggests that they were once separate prokaryotes that were endocytosed into a eukaryotic cell, which explains why mitochondria are encased in a lipid bilayer and have their own genome comprised of mitochondrial DNA (mtDNA) [69] allowing them to synthesize their own proteins [70]. Despite having several independent features, mitochondria still depend on the cellular nucleus to complete vital biosynthetic functions. The crosstalk between the nucleus of a cell and the mitochondria is essential in ensuring proper mitochondrial function [59, 71]. The endosymbiotic origins and semi-autonomy of mitochondria may thus share critical insights into how mitochondria can be isolated from one cell and transferred into another. Cells can donate their mitochondria or mtDNA to damaged cells, rescuing the recipient’s metabolic function [72]. This phenomenon, however, is only observed when there is a significant level of damage to the host mitochondria or mtDNA. However, if there were mutations in the host cell’s mtDNA, the transfer did not occur [73]. This limitation is problematic, as the damage to the mitochondrial viability within the cells is often to such an extent that endogenous mitochondria are not enough to rescue tissue function.

### Mitochondrial intake into cells

In 1982, Clark and Shay published ‘Mitochondrial Transformation of Mammalian Cells’, where they developed the first method to transplant mitochondria from a xenogeneic source into another cell. Clark and Shay first isolated mitochondria that showed resistance to the antibiotics chloramphenicol and efrapeptin. They then co-incubated the antibiotic-resistant mitochondria with cells that showed no antibiotic resistance. The cells took in the mitochondria, and the antibiotic resistance from the mitochondria transferred to the cells [74]. In 2007, mitochondria isolated from rat cells were successfully incorporated into compromised human mesenchymal stem cells (MSCs), improving metabolic function [75]. In 2009, McCully *et al.* isolated mitochondria from healthy rabbit heart tissue and injected them into ischemic heart tissue, reducing infarct size and demonstrating the effects of mitochondrial transplantation on the tissue scale. They also showed that injecting frozen or compromised mitochondria did not have the same beneficial effects, indicating that mitochondrial viability is a critical parameter in the transplantation process [76].

**Figure 2.**
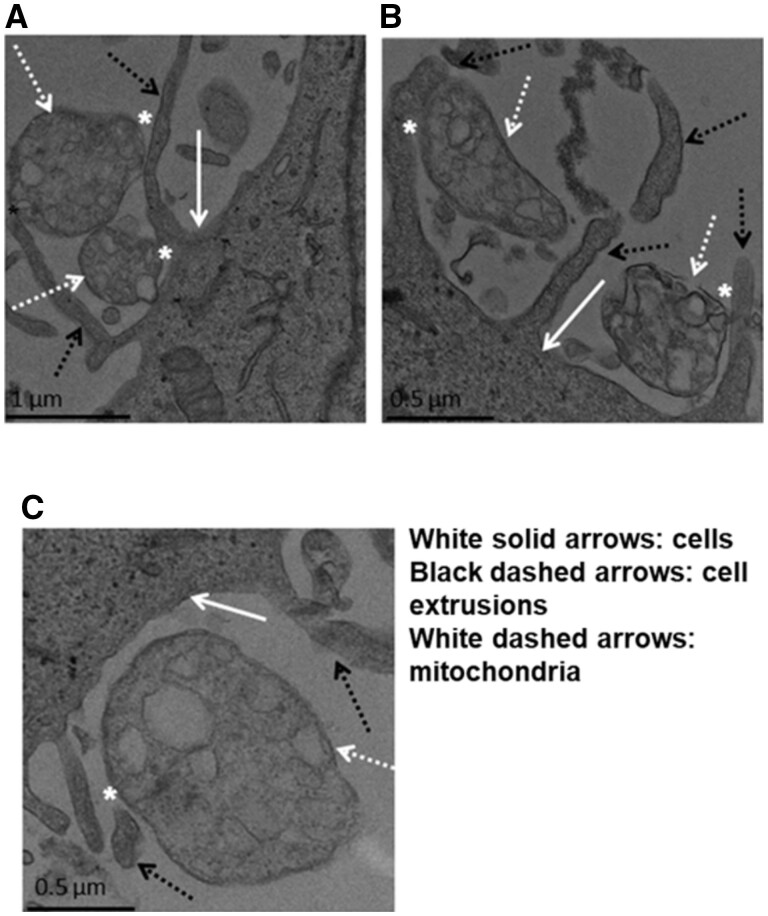
Mitochondrial intake into host cells. Adapted from Kesner *et al.* [77].

As demonstrated in numerous studies, mitochondria can be incorporated into cells without the need for any transfection reagents or intervention. While the exact mechanisms of this process are still being investigated, current studies illustrate the possible involvement of macropinocytosis and caveolae-dependent endocytosis (Fig. 2). This dependency was assessed by pharmacologically inhibiting macropinocytosis and caveolae-dependent endocytosis, impeding mitochondrial uptake into cells [77]. A later study showed that macropinocytosis inhibitors but not clathrin-mediated endocytosis impeded mitochondrial incorporation [78]. Live fluorescence imaging involving DsRed1-labeled exogenous mitochondria intake into GFP-expressing recipient cells has shown the engulfment of mitochondria via cellular extensions, further indicating macropinocytosis or macropinocytosis-like mechanisms [77, 78].

Interestingly, studies involving pharmacological blocking of actin polymerization have also indicated that mitochondrial intake into cardiomyocytes occurs via actin-dependent endocytosis. Cardiomyocytes incubated with an actin polymerization inhibitor exhibited a significant reduction in exogenous mitochondrial intake [79]. Transfer of mitochondria between cells has also been demonstrated to occur via tunneling nanotubes and non-adherent actin-based cytoplasmic extensions that facilitate the transfer of signals and cellular contents. Additionally, mitochondrial transfer has been shown to occur via extracellular vesicles [80, 81]. For a comprehensive review of the mechanisms of mitochondrial transfer, the authors of this paper suggest ‘Mitochondria know no boundaries: mechanisms and functions of intercellular mitochondrial transfer’ by Torralba *et al.* [82]. There is compelling evidence of various separate mechanisms by which exogenous mitochondria are taken into host cells, suggesting that the method of cellular intake may depend on cell type, method of delivery, mitochondrial sourcing or other factors, thus necessitating further research.

### Mitochondria as a therapeutic agent

Following the promising results of *in vitro* experiments, mitochondrial transplantation research quickly transitioned towards *in vivo* work. Using a direct injection technique, McCully *et al.* injected autologously derived isolated mitochondria into ischemic rabbit hearts before reperfusion [83]. The injection increased mechanical function as soon as 10 min after, resulting in significant increases in ATP production after 2 days, with benefits being maintained 21–28 days post-reperfusion. The discovery that cardiomyocytes could incorporate exogenous mitochondria was made after only two hours. This group subjected rabbit hearts to thirty minutes of focal ischemia. They then either applied autologously derived mitochondria via direct injection or by vascular delivery through the coronary artery. The results of this study illustrated a wider distribution of mitochondria via vascular delivery and a more condensed spread of mitochondria via direct injection. Both methods resulted in a large number of mitochondria depositing within the interstitial fluid. However, there was still some level of uptake from the cardiomyocytes. Post-reperfusion, infarct size was reduced, and preservation in the elastic properties of the cardiac tissue was observed [84, 85].

In these studies, key inflammatory markers were either reduced or not significantly higher [58]. Specifically, IL-6, IL-10, macrophage inflammatory protein 1 and C-reactive protein levels were shown to be substantially reduced compared to untreated regional ischemic tissue. This observation is critical in two facets: first, in further confirming the safety of clinical uses of mitochondrial transplantation, as previous studies have indicated that extracellular mtDNA triggers an immune response that can adversely affect wound healing and progress into tissue damage [86–88]. Second, the reduction of pro-inflammatory markers suggests an immunomodulatory effect of mitochondrial transplantation.

Numerous studies have been conducted on attenuating tissue damage due to injury by utilizing mitochondrial transplantation. However, there are still extensive gaps in current scientific knowledge. This review will emphasize two critical avenues of investigation that remain to be explored. First, how can mitochondrial transplantation affect wound healing and host–biomaterial dynamics? Can this technique help shift biomaterial implantation dynamics away from fibrous encapsulation and towards tissue–device integration? Second, the scope of most investigations into mitochondrial transplantation has mainly been limited to either broad-scope, macro-scale effects on the target tissue or narrow-scope focused research on the target parenchymal cells (i.e. focusing solely on cardiomyocytes on myocardial infarction models and not on any potential effect on off-target immune or support cells). This scientific knowledge gap leaves myriad opportunities for potential new applications of mitochondrial transplantation, as well as opportunities to elucidate the unclear underlying mechanisms behind the reported benefits of mitochondrial transplantation.

### Mitochondrial sourcing and dosage

Mitochondria have been isolated from a variety of different sources for the purposes of mitochondrial therapies. Mitochondria have been isolated from allogeneic, autologous and, in some cases, xenogeneic tissues [75, 89, 90]. Pectoralis major muscle, soleus muscle, gastrocnemius muscle and liver biopsies have been used for whole tissue isolation due to their high mitochondrial content, with 3–5 mg of tissue needed for suitable mitochondrial numbers [58, 89]. Various cell lines have also been used for mitochondrial sourcing, including stem cells (primarily mesenchymal and umbilical), H9c2 cells and L6 cells [89].

The dosages or number of mitochondria used for therapeutic purposes also vary greatly, with mitochondrial numbers between 1 × 10^6^ and 1 × 10^9^ being injected for *in vivo* studies [91]. Some publications report mitochondrial dosages in terms of the mass of mitochondrial content delivered, varying from 10 to 1500 µg [92–94]. Certain dosages for systemic applications were normalized to body weight. For example, a liver toxicity model injected 0.2 or 0.4 mg/kg mitochondria once every day for 7 days [95]. It is important to note that an optimum dosage was not identified throughout all of these publications, even in ones that tested multiple dosages.

For more examples, see Table 2. For a comprehensive review of mitochondrial sourcing and isolation procedures, the authors of this paper recommend ‘Requirements for successful mitochondrial transplantation’ by Kubat *et al.* [89]. There is still a great need for investigation into the advantages and disadvantages of various sources for mitochondria so that a gold standard source may potentially be found. Additionally, even the optimal dosages or mitochondrial number remains controversial, even for similar applications or target tissues. Further research must be conducted for mitochondrial therapies to be standardized and brought closer to real therapeutic use in the clinic.

**Table 2.** Overview of studies investigating mitochondrial transplantation

Disease/injury	Mitochondrial sourced from	Implanted into	Delivery via	Outcome	Reference
Acute lung injury	Bone marrow-derived stromal cells (mouse and human)	Alveolar epithelia	Intranasal instillation	Increased alveolar ATP and abrogated ALI pathologies	Islam *et al.* [96]
Blood-perfused regional ischemia	Autologous muscle cells	Myocardial cells	Injection of mitochondria-containing respiration buffer	Decreased myocyte necrosis and post-ischemic function	Masuzawa *et al.* [83]
Ischemia	Mesenchymal multipotent stroma cells	Cortical neurons and astrocytes	Co-culture	Improved cell viability	Babenko *et al.* [97]
UV light damage	PC12 (pheochromocytoma cell line)	PC12	Co-culture	mtDNA transfer	Wang and Gerdes [98]
Infertility	Oogonial precursor cells	Human oocyte	Intracytoplasmic injection	Increased *in vitro* fertilization success	Oktay *et al.* [99]
Ischemia	Bone marrow-derived mesenchymal stem cells	H9c2	Co-culture	Reduced apoptosis process	Han *et al.* [100]
Transient focal cerebral ischemia	Mouse cortical astrocytes	Peri-infarct complex	Direct injection or autologous secretions	Promoted adjacent neuronal survival and plasticity after injury transfer	Hayakawa *et al.* [102]
Parkinson’s disease	PC12 (pheochromocytoma cell line); human osteosarcoma cybrids	PD rats/brain neurons	Local injection at medial forebrain bundle	Improved locomotive activity and attenuated deterioration of dopaminergic neurons	Chang *et al.* [90]
Acute myocardial infarction	Autologous porcine muscle cells	Myocardial cells	Injection of mitochondria-containing respiration buffer	Improved cell viability post-ischemia	Kaza *et al.* [192]
Spinal cord injury to L1/L2 vertebra	PC12, syngeneic muscle cells	Brain macrophages, endothelium, pericytes, glia	Microinjection at mediolateral gray matter	Maintenance of acute mitochondrial bioenergetics enhanced behavioral recovery	Gollihue and Rabchevsky [85]
Non-alcoholic fatty liver disease	HepG2 hepatocellular carcinoma cell line	Multiple tissues	Intravenous injection	Decreased lipid content and restored cellular redox balance	Fu *et al.* [103]
Sciatic nerve crush	BHK-21 (baby hamster kidney) cells	Epineurium	Direct injection	Decreased ROS production and improved cytoskeletal structure of nerve	Kuo *et al.* [168]
Schizophrenia	Epstein-Barr virus transformed lymphocytes or rat brain	Intracerebral tissue	Co-culture (*in vitro*), direct intracerebral injection (*in vivo*)	Increased mitochondrial function, reduction of symptoms	Robicsek *et al.* [104]
Traumatic brain injury	Cortical neurons	Hippocampal neurons	Add in medium	Enhanced neuroregeneration	Chien *et al.* [105]
Acetaminophen-induced liver damage	HepG2 hepatocellular carcinoma cell line	Multiple tissues	Intravenous injection	Increased hepatocyte energy supply and reduced oxidative stress	Shi *et al.* [106]
Skin graft	Allogenic and syngenetic mice muscle cells	Muscle cells/skin cells	Direct injection	Absence of immune response or DAMPs	Ramirez-Barbieri *et al.* [107]
Lipopolysaccharide-induced depression	Mice hippocampal tissue	Mice hippocampus tissue	Intravenous injection	Decreased neuroinflammation and oxidative stress, increased neurogenesis and brain-derived neurotrophic factor expression	Wang *et al.* [101]
Breast cancer	143B osteosarcoma cybrids containing healthy mitochondrial DNA	MCF-7 breast cancer cells	Co-culture (both conjugated with Pep-1 and without)	Inhibition of cancer cell proliferation, induction of caspase-independent and AIF-mediated cell apoptosis, increased chemotherapeutic sensitivity	Chang *et al.* [108]
Acute kidney injury	Murine muscle tissue biopsy	Murine kidney undergoing ischemia–reperfusion	Anterograde injection via the renal artery	Lowered plasma creatinine, increased glomerular filtration rate, lowered blood urea nitrogen, increased urine output	Doulamis *et al.* [109]
Acute limb ischemia	Murine muscle biopsy, human cardiac fibroblasts	All muscles of the hindlimb	Direct injection into muscle	Increased ATP content, lowered damage in histology images, improved stance time, lowered dragging of limb, lower infarct size	Orfany *et al.* [91]
Cardiogenic shock following ischemia–reperfusion injury	Autologous muscle biopsy	Pediatric heart tissue	Direct injection into cardiac tissue	Increased rates of successful separation from extracorporeal membrane oxygenation (ECMO). Enhanced ventricular strain of heart tissue	Guariento *et al.* [110]
Cerebral ischemia–reperfusion injury	mNSC and N2a cell lines	Rat cerebral infarct area	Direct injection into cerebral infarct area	Improved neurobehavioral deficits. Reduced infract size of MCAO rat. Lowered ROS and apoptosis levels	Xie *et al.* [111]
Tendinopathy-mediated inflammation	Umbilical cord-mesenchymal stem cells (UC-MSCs) L6 cells	Tenocytes treated with TNF-α Collagenase-treated rat tendons	Co-incubation with tenocytes Direct injection into the tendon	Downregulated mitochondrial fission factors ATP restoration Upregulated fusion factors Suppressed NF-κB signaling Reduced pro-inflammatory marker (IL-1β and IL-6) levels Inhibited apoptosis Increased collagen content Reduced tendon swelling, MMP1 and TNC levels	Lee *et al.* [93]
Spinal cord injury	Soleus-derived allogeneic mitochondria	T10 spinal cord after application of aneurysm clip	Direct injection into the injury site	Recovery of locomotor and sensory functions Reduced dynamin-related protein 1 and severity of demyelination Reduced apoptosis and inflammatory markers	Lin *et al.* [112]
Mesenchymal stem cell culture for tissue engineering	Allogeneic human donor adipose-derived stem cells (ADSCs)	ADSCs	Co-culture	Higher ATP content, altered secretome yielding DNA replication and cell division Enhanced proliferation, migration and differentiation Prolonged cell survival, engraftment and horizontal transfer to host cells Improved skin repair in *in vivo* rat skin-defect model	Yao *et al.* [113]
Neurological damage after resuscitation from cardiac arrest	Allogeneic rat brain tissue Allogeneic pectoral rat muscle	Brain following resuscitation after 10 min of cardiac arrest	Intravenous infusion	Improved survival from 55% to 91% Increased neurological recovery Improvements in metabolism, cerebral blood flow and lung edema	Hayashida *et al.* [114]

### Current benefits and promising results of mitochondrial therapies

Mitochondrial transplantation has been investigated as a solution for various ailments, ranging from heart attacks and strokes to mental health disorders such as lipopolysaccharide-induced depression [76, 84, 101, 102]. Below is a summary of research, *in vivo* and *in vitro*, involving mitochondrial transplantation [59, 108].

## The potential of mitochondrial therapies within the host–biomaterial response and healing continuum

**Figure 3.**
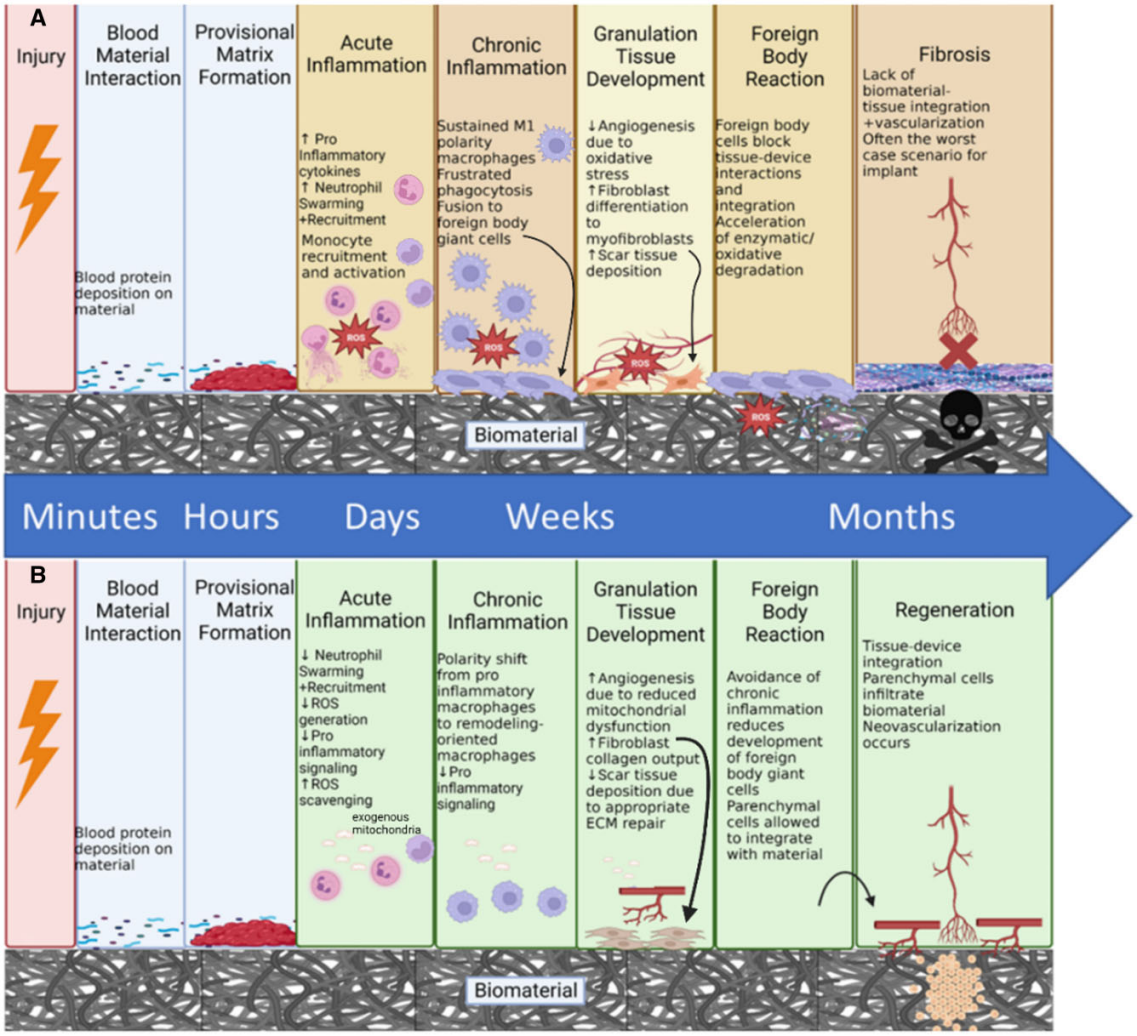
Unsuccessful resolution of key phases leading to repair (**A**). Potential for mitochondrial therapies to aid resolutions of phases and to drive regeneration (**B**). Created with BioRender.com.

Based on the findings in the studies enumerated in Table 2 and viewing them through the lens of wound healing and biomaterial integration shown in Table 1, numerous exciting results and areas warrant further investigation that could yield immense promise as tissue engineering and regenerative medicine therapeutics are explored and developed. The following paragraphs highlight these findings and promising avenues in the context of host–biomaterial interaction and wound healing stages. It also indicates the points of opportunity wherein mitochondrial dysfunction plays a causative effect on the unsuccessful resolution of the wound healing phase. Introducing a mitochondrial transplantation-based therapy could potentially help shift the continuum away from repair and towards regeneration (Fig. 3).

### Potential for mitochondria in the injury phase

Injury initiates the host response continuum and occurs as a consequence of the surgery necessary to implant a given biomaterial [115]. The response to initial injury is affected by several parameters, the extent of the incision, loss of membrane structures, the extent of cellular necrosis and blood loss from the surrounding tissues [2]. As aforementioned, it has been proven many times that mitochondrial transplantation alleviates the effects of ischemia and reperfusion and restores native tissue function [58, 76, 83, 116]. This restoration indicates a potential avenue by which mitochondrial therapies may be applied. Studies involving injecting isolated mitochondria into the heart in early ischemia, before reperfusion, showed cardioprotective effects, reducing damage post-reperfusion [76]. Thus, by transplanting healthy mitochondria into the surgery site, it may be possible to mitigate the damage induced by surgery. This simple solution may be a novel approach for limiting the tissue damage involved in biomedical device implantation, which may, in turn, have cascading effects down the host response continuum, ameliorating unwanted inflammation, thrombosis and fibrous capsule development, while expediting ECM regeneration and angiogenesis. Further research should be done regarding the timing of mitochondrial therapies to see if introducing exogenous mitochondria into the wound site prior to the induction of inflammation could have protective effects. The findings of such investigations could reveal whether mitochondrial therapies are not just useful for treatment after the development of pathological conditions but could be used as a prophylactic measure.

### Potential for mitochondria in the BMI and provisional matrix formation

The BMI phase plays a dual-sided role in terms of implanted biomaterials. In the context of wound healing, blood protein buildup is known as provisional matrix formation, as it provides structure and many of the materials necessary to close a wound [117]. However, in the context of biomaterial implantation, thrombosis can block the device–tissue interface, resulting in decreased implant efficiency, and leading down the road to the organization with fibrous tissue development, thus resulting in fibrosis [4, 118]. Therefore, blood clot formation around the biomaterial, or thrombosis, is another critical parameter to be limited in the implantation of biomaterials. Mitochondrial dysfunction initiates a vicious cycle, initiating thrombosis, while thrombosis causes ROS to be produced [119]. Oxidative stress has been implicated in altering metabolites in such a way that prostacyclin, an eicosanoid lipid molecule that inhibits platelet activation, is inhibited while increasing levels of prostaglandin H_2_, which may lead to adverse effects such as vasoconstriction and thrombosis [120, 121]. This phenomenon demonstrates an incentive to preserve mitochondrial viability immediately after an injury.

Previous beliefs limiting the role of mitochondria in thrombotic signaling to merely anabolic purposes have since been found to be incorrect [122]. Mitochondria are present in small numbers in platelets and are vital in non-ATP thrombotic signaling and platelet apoptosis signaling [123–125]. In a hyperglycemia model, increased ROS production was found to have a potentiating effect on both platelet aggregation and the release of platelet-derived growth factor AB [126]. Aberrant ROS overproduction by mitochondria has been shown to underlie the activation of platelets, indicating that mitochondrial dysfunction may increase platelet aggregation [127]. However, pharmacological blocking of the electron transport chain has also decreased granule secretion and platelet aggregation [128, 129]. These findings suggest that there is a delicate balancing act in terms of the role of mitochondria in preventing thrombosis. On the one hand, it is vital that mitochondria remain viable and ROS production is kept at a minimum. Yet, the loss of mitochondrial membrane potential is an essential step in the apoptosis of platelets [130]. It is critical that more research is done into the effects of mitochondrial transplantation on thrombosis, as the presence of transplanted mitochondria may be an added benefit or a potential limiting factor depending on how this delicate balance is struck. It may be beneficial in tissue engineering to introduce cells of the same type as the native tissue, seeded with transplanted mitochondria, into the healing implant site to mitigate the ROS damage that inevitably occurs during platelet apoptosis, avoiding an increase in platelet mitochondria count. This reliance on the loss of mitochondrial membrane potential in platelets may depend on future findings, making a compelling case against the previously discussed direct injection method of mitochondrial transplantation.

### Potential for mitochondria in reducing inflammation

Inflammation is generally described as a biological response intended to defend the body from anything it deems foreign. While inflammation is vital to protecting cells and tissues from damage, as well as marking, sequestering and killing pathogens, if it does not cease appropriately, it can lead to chronic inflammation or even organ failure [131]. An overabundance of pro-inflammatory cytokines has implicated the progression of diseases and other health concerns, including, but not limited to, cancer, Parkinson’s disease, obesity and diabetes [64, 132, 133]. Inflammation and mitochondrial dysfunction are intimately linked, and ROS is present in several of the aforementioned inflammatory diseases [134–137]. It has become clear recently that mitochondria play a role in both intracellular danger-signaling and extracellular danger signaling, setting off the chain of events that leads to an inflammatory response. If cellular damage has reached a point where the cell can no longer produce the energy required to sustain itself or if it can no longer maintain the delicate balance of membrane potentials necessary to maintain homeostasis, the body initiates an immune response. Mitochondrial Outer Membrane Permeabilization (MOMP) is one of these warning signs [138]. It represents a nearly universal point of no return in terms of cell death, leading to a cascade of signal transductions, with the endpoint being apoptosis or regulated necrosis [139]. In terms of extracellular danger signaling, there are various products that mitochondria can emit that elicit an immune response, including mtDNA, ROS and specific mitochondrial proteins [140, 141]. These effects are named mitochondrial damage-associated molecular patterns (mDAMPs). Recent studies have linked mDAMPs, including mtDNA, NFPs and various mitochondrial lipids, to massive neutrophil responses, damaging multiple organs and systemic inflammatory response syndrome (SIRS) [140].

### Extracellular mtDNA in the immune response

In cases involving trauma, fractures, burns and organ damage, after extensive cell necrosis, free circulating mtDNA can be found in the bloodstream [140, 142, 143]. Because of the prokaryotic origins of mitochondria, mtDNA is very similar to bacterial DNA. mtDNA, like bacterial DNA, contains unmethylated CpG regions. These regions are detected by toll-like receptor 9 (TLR9), which is an endosomal pattern recognition receptor expressed in numerous immune cells [144]. Extracellular mtDNA has been shown to recruit neutrophils and activate them via TLR9 [140]. These effects are also observed in the pathogenesis of chronic illnesses. In chronic illnesses, mtDNA in the bloodstream sets off a cell-autonomous TLR9-dependent signaling cascade leading to the release of interleukin-1β, a critical pro-inflammatory cytokine, as well as the release of Interleukin-6, which can be either pro-inflammatory or anti-inflammatory depending on the context [145–147]. In the case of healthy cells, this response can often be mediated by autophagy, where the critically damaged mitochondria are intercepted and degraded before they can release their DNA, avoiding the activation of inflammasomes. The response also works by initiating the degradation of mtDNA within endosomes, which prevents TLR9 activation [148, 149]. As well as cell danger signaling, this release of mtDNA has been observed to be implemented by certain eosinophils to trap foreign matter in the ECM [150]. When neutrophils become activated via interleukin-5 or interferon γ, they release their mtDNA in a ‘catapult-like’ manner to ensnare foreign objects in a phenomenon dubbed NETosis [151]. When functioning correctly and attenuating properly, this phenomenon helps the body wall off antigens and defend the surrounding tissue. However, the buildup of these extracellular traps can lead to excessive scar tissue or fibrous capsule envelopment in biomedical implants [43, 152].

### NFPs in the immune response

Similarly to mtDNA, NFPs, released from the cell during necrosis, are produced by the mitochondria and show strong resemblances to bacterial products. NFPs have been implicated in several harmful immune responses, including but not limited to organ damage, post-trauma SIRS and smoking-triggered lung emphysema [140, 142, 153]. These immune responses occur when NFPs bind to formyl peptide receptor-1 on the phospholipid bilayer of neutrophils. This binding stimulates intracellular calcium ion signaling and the activation of mitogen-activated protein kinases (MAPKs). MAPKs operate as powerful chemoattractants and activators of degranulation, where cells release cytotoxic, antimicrobial or other molecules such as histamines. NFPs binding to receptors also trigger oxidative bursts in some cells, where cells release ROS. As explored later, this can lead to a vicious, inflammatory cycle [16].

### Reactive oxygen species in the immune response

Roughly 1–3% of the molecular oxygen used during oxidative phosphorylation fails to reduce completely, generating superoxide anions. When mitochondria become compromised by damage or mutation, this percentage can increase to a dangerous level, setting off a chain reaction, as ROS can further damage mitochondria or mtDNA. Since mtDNA is prokaryotic in origin, it does not weave into a protective structure via histones. It does not have the enzymatic safety and repair mechanisms afforded to the cell’s eukaryotic nuclear DNA [64]. The most significant mitochondrial ROS sources are ubiquinone and NADH dehydrogenase (complex 1). The enzyme superoxide dismutase typically converts superoxide to hydrogen peroxide. However, when hydrogen peroxide reacts with transition metals, they form hydroxyl radicals. These hydroxyl radicals are also a key mediator of cellular damage. Superoxide can also react with nitric oxide to form peroxynitrite in a reaction that occurs at three times the rate of superoxide dismutation by superoxide dismutase. At physiological pH, peroxynitrite can exist in an activated ‘hydroxyl radical-like’ stage, diffusing over areas that can encompass multiple cells, causing damage by oxidizing lipids, proteins and nucleotides [154]. At sites of inflammation, hydrogen peroxide can be converted into hypochlorous acid by the enzyme MPO, which is expressed by neutrophils. The detection of these free radicals by cells can lead to several pathophysiologically significant outcomes, including the activation of redox-sensitive transcription factors like hypoxia-inducible factor 1-α and nuclear factor-κB, the activation of pro-inflammatory cytokines and inflammasomes [155].

Mitochondrial ROS has been shown to contribute to the lipopolysaccharide-mediated production of the highly pro-inflammatory cytokines interleukin-1β, interleukin-6 and tumor necrosis factor-α. In terms of inflammasome activation, high levels of ROS activate NLRP3, where it recruits ASC and procaspase-1, mediating proteolytic cleavage, resulting in the activation of caspase-1, along with interleukin-1β and interleukin-18. While mitochondrial ROS activates these products, healthy levels of autophagy and mitochondrial autophagy (mitophagy) can significantly reduce the activity of inflammasomes and the activation of these cytokines. Autophagy and mitophagy regulate intracellular oxidative stress by degrading dysfunctional mitochondria before they can start overproducing ROS. Experimental evidence suggests that macrophages isolated from mice with defective autophagy proteins LC3B and ATG16L1 had significantly higher levels of NLRP3 inflammasome activation and higher amounts of interleukin-1β and interleukin-18 production [133].

Studies show that elevated levels of ROS disrupt the body’s natural calcium ion signaling mechanisms [156]. Endothelial barrier integrity is dependent on intracellular calcium concentrations. Increases in intracellular calcium concentrations can lead to inter-endothelial cellular gaps, vascular hyperpermeability and calcium-calmodulin-dependent myosin light chain kinase activation. This calcium-calmodulin-dependent myosin light chain kinase induces the reorganization of cellular actin filament reorganization, altering endothelial cell shape [157]. Increases in intracellular calcium concentrations were found to be linked with the production of hydrogen peroxide.

Conversely, increases in hydrogen peroxide caused increases in intracellular calcium concentrations [158, 159]. These effects culminate in high vascular permeability, a significant indicator of inflammation, implicating mitochondrial ROS production with sterile inflammation. Sterile inflammation is the body’s immune response in the absence of a foreign antigen and is typically triggered by a large volume of cell death without proper autophagy.

### Mitochondrial dysfunction increases sensitivity to pro-inflammatory cytokines

IL-1β is a major pro-inflammatory cytokine that, upon detection by a cell, can modulate the production of several chemokines, including IL-8. IL-8 is a chemokine responsible for inducing the chemotaxis of neutrophils to the site of infection. The pathogenesis of osteoarthritis strongly resembles what is considered the worst-case outcome in wound healing. Rampant and unresolved inflammation leads to a degradation of the ECM, cell death and further pro-inflammatory signaling. Recent findings have shown decreased respiratory chain integrity in osteoarthritic chondrocytes compared to healthy chondrocytes [160]. In an *ex vivo* model of normal human chondrocytes, when oxidative stress was induced via mitochondrial inhibitors antimycin A and oligomycin, a dose-dependent increase in IL-8 mRNA expression was found. The mitochondria-deficient chondrocytes’ IL-8 production in response to IL-1β was assayed, and a significant increase in the cell’s sensitivity to IL-1β was found, demonstrating that a cell’s response to inflammatory cytokines can be increased by an overproduction of ROS [161]. This heightened IL-8 signaling due to oxidative damage from dysfunctional mitochondria would explain the continued chemotaxis of activated neutrophils to the cartilage, causing sustained immune attack and further inflammation [162].

Additionally, the heightened sensitivity to Il-1β throughout the microenvironment would maintain pro-inflammatory phenotypes in cells within and entering the cartilage [161, 163]. Thus, not only do cells with dysfunctional mitochondria produce higher levels of inflammatory signals, but they also increase surrounding cells’ susceptibility to these signals. The positive feedback loop that causes and maintains chronic inflammation in damaged tissues has been discussed previously. These findings suggest that mitochondrial therapies may offer an intervention to cease this positive feedback loop, preventing chronic inflammation and allowing regeneration of the affected area.

### Mitochondrial transplantation ameliorates inflammation

Parkinson’s disease is a chronic, neurodegenerative disease characterized by a loss of motor function. Recent studies have found strong evidence implicating mitochondrial dysfunction, oxidative stress and inflammation within the substantia nigra of the brain to the pathogenesis of Parkinson’s [164]. Injection of allogeneic and xenogeneic isolated mitochondria into the substantia nigra was performed to determine if the oxidative stress and neuroinflammation would reduce. Pep-1, an amphipathic peptide carrier that has demonstrated the ability to transverse cell membranes, was appended to the isolated mitochondria to promote cell integration. Mitochondria were isolated from allogeneic PC12 cells, as well as xenogeneic human osteosarcoma cells. They were then assayed for viability, as mitochondrial membrane potential is vital for transplantation [165]. The mitochondria were then injected into rat PC12 cells and into the medial forebrain bundle for *in vivo* analysis. The research group assayed cell viability, neurite outgrowth and oxidative stress. The findings of these experiments suggested that both the xenogeneic and allogeneic mitochondria produced positive results, but the effects were more profound in allogeneic transplantation. As a result of mitochondrial injection into the substantia nigra, a significant reduction in interleukin-5, interleukin-17a and interleukin-10, all pro-inflammatory cytokines, was observed. The group also found significant decreases in ROS production and therefore reduced cell apoptosis levels. They also found the rescue of native mitochondrial function as well as rescued cell respiration and healthy metabolism [90]. In an LPS-induced depression study, researchers observed a significant decrease in the inflammatory activation of astrocytes and microglia and a steep reduction in IL1-β, TNF-α and COX-2 expressions. Notably, there was also a decrease in ROS production [101]. These results show a promising new future for the reduction of inflammation via mitochondrial transplantation that can save native cells as well as reduce the amount of inflammatory signaling within the affected area. Recent studies further corroborate these findings. In both *in vitro* and *in vivo* studies of tendinopathy, mitochondrial transplantation into cells undergoing immune stress stimulated by TNF-α decreased intracellular ROS generation, inhibited apoptosis, downregulated expression of IL-1β and IL-6 and decreased the levels of phosphorylation of NF-κB [93].

Additionally, inflammatory markers were significantly reduced in a recent study investigating the efficacy of mitochondrial therapies for treating tendinopathy. This study found that when healthy exogenous mitochondria were introduced to inflamed tenocytes, NF-κB and MMP1 were lowered, resulting in a subsequent reduction in pro-inflammatory cytokines IL-1β and IL-6, and inflammation-mediated degradation of the tendon tissue *in vitro*. These findings were further confirmed in *in vivo* investigations wherein exogenous mitochondria were incorporated into the inflamed Achilles tendon of rats, reducing TNF-α, IL-1β and IL-6. Notably, in this study, TNF-α was reduced to baseline regardless of mitochondrial dosage [93].

In a polymicrobial sepsis mouse model, mice treated with intravenous injections of exogenous mitochondria displayed lowered systemic inflammation, higher survival rates, increased bacterial clearance and attenuated organ damage. The mice were subjected to cecal ligation and puncture to induce sepsis. Then, exogenous mitochondria isolated from allogeneic muscle biopsies were injected into the tail veins of the septic mice. The mice that received systemic replenishment of mitochondria demonstrated significantly lower systemic levels of IL-6, IL-1β and JE (murine monocyte chemoattractant protein homolog). Histological and biochemical evaluations of tissue damage indicated that leukocyte infiltration and edema were significantly reduced upon treatment with mitochondria [166].

Further, in another sepsis study, pro-inflammatory stimulated microglia were treated with exogenous mitochondria. LPS, IFN-γ and IL-4/IL-13 were used to induce different phenotypes of BV2 microglial cells and drive them toward M1 pro-inflammatory phenotypes. Treatment with exogenous mitochondria lowered pro-inflammatory cytokine TNF-α, IL-6 and IL-1β release while increasing anti-inflammatory cytokine TGF-β and IL-4 release. Microglia treated with mitochondria expressed higher M2 anti-inflammatory markers, signaling a shift from M1 predomination to a restorative M2 predomination in the septic microenvironment. *In vivo* evaluations of neurological deficits indicated that intracerebroventricular injection of exogenous mitochondria in mice undergoing cecal ligation and puncture-induced sepsis ameliorated loss of motor control and body weight due to neuroinflammation [167]. These recent findings shed critical insight into the potential immunomodulatory effects of mitochondria-based therapeutics.

### Mitochondrial transplantation and prevention of immune response positive feedback loops

In a heart-ischemia model, mitochondrial transplantation decreased infarct size, improved cardiac muscle function and upregulated chemokines and protein expressions important in cardiomyocyte function and survival. The upregulation of these signals resulted in a reduction of both apoptosis and necrosis in ischemic tissue [83]. Cerebral ischemia has been ameliorated via the delivery of isolated mitochondria after a stroke was induced in rats. It was also found that there was an increase in cell-survival signals produced after 24 h of mitochondria delivery [102]. Mitochondria were shown to transition from a damaged anaerobic state to aerobic respiration after introducing healthy mitochondria. This rescue of metabolic function was indicated by a 300% increase in ATP production and a significant reduction in the lactate output of the damaged cells [72]. In addition to increased ATP production, ROS levels have also been investigated post-mitochondria transfer. In a sciatic nerve crush model in rats, ROS production was significantly reduced after mitochondria were injected into the epineurium, leading to a better recovery rate of the injured nerves and the denervated muscles [168]. These findings show that mitochondrial transplantation can alleviate inflammatory responses and prevent them from happening in the first place. Either by preventing necrosis during trauma or injury or by reducing the levels of ROS production, putting an early halt to the inflammation pathways discussed earlier.

### Potential for mitochondria in promoting granulation tissue development

The next step in the wound healing process is the formulation of granulation tissue, named for its granular, soft and pink appearance. This process is characterized by the predominance of monocytes and macrophages, followed by the proliferation of fibroblasts and vascular endothelial cells. In this phase, healing inflammation begins, and the body shifts from fighting off infection to repairing and remodeling the missing or damaged tissue. Proteoglycans predominate in the early stages of granulation tissue development. Collagen then becomes the primary component of ECM regeneration [169, 170].

Fibroblasts are responsible for the formation of the granulation tissue ECM. Transforming growth factor (TGF)-β, derived from platelets, presents significant signaling events necessary for the activation and proliferation of fibroblasts [171]. An investigation showed that rabbit wounds treated with ATP vesicles had a significantly higher expression of monocyte chemoattractant protein-1 (MCP-1) and other signaling molecules than controls not treated with ATP [172]. In wound healing, MCP-1 stimulates collagen expression through the upregulation of TGF-β in stimulated fibroblasts, thus amplifying collagen fiber and ECM formation [172]. Additionally, collagen production and secretion are reduced under oxidative stress conditions, leading to improper ECM repair and prolonging wound healing [173]. A potential therapeutic opportunity for exogenous mitochondria could be to reduce the oxidative stress experienced by fibroblasts, possibly allowing for improved collagen secretion and other anabolic processes.

Moreover, fibroblasts will differentiate into myofibroblasts upon incomplete or impaired remodeling of the wound or implant environment. Myofibroblasts secrete higher levels of disorganized, densely packed collagen and have characteristics of smooth muscle cells to pull the wound environment closed prematurely [174]. The dense, disorganized collagen becomes scar tissue which acts as a plug for the injury site, walling it off from the rest of the body. Myofibroblast differentiation occurs through various pathways, including pro-inflammatory signaling from macrophages and conditions of oxidative stress [175, 176].

New findings have indicated that transplantation of polymer-functionalized mitochondria into TGF-β stimulated fibroblasts prevented fibroblast to myofibroblast transition by preventing the shift of fibroblasts to glycolytic metabolism. These effects then reduced the numbers of fibroblasts differentiating into myofibroblasts, prevented excessive fibroblast infiltration and altered the expression and release profiles of the fibroblasts away from pro-fibrotic [177]. These findings present great promise in shifting granulation tissue development towards regeneration despite the presence of pro-fibrotic signals such as TGF-β. It should be noted that the uptake of mitochondria into host fibroblasts in this study was facilitated via a dextran-triphenylphosphonium coating, showing improved cellular uptake compared to uncoated mitochondria, suggesting that polymer functionalization via lipophilic, cationic ligand coating may ease intake [178].

Another vital step in the granulation tissue development phase is the induction of angiogenesis. In this step, the damaged vasculature is regenerated, and additional blood flow is supplied to the area to support remodeling efforts by the support and immune cells. The constant influx of oxygen and nutrients into the microenvironment is vital for complete healing and prevention of fibrosis. Evidence has indicated that angiogenesis is impaired by mitochondrial dysfunction. The ensuing oxidative stress and lowered metabolic capabilities of the microenvironment do not allow for the formation of new blood vessels [179]. Additionally, findings have suggested that vascular endothelial growth factor, a vital signal for inducing angiogenesis, is suppressed, despite hypoxia in the damaged tissues [180]. Thus, not only does mitochondrial dysfunction directly impair angiogenesis, but it also decreases the output of growth factors needed to recruit surrounding cells and stimulate blood vessel formation.

By reducing the oxidative damage and pro-inflammatory conditions experienced in the microenvironment, mitochondrial therapies could prove to be a valuable asset in restoring the ECM and vasculature of the wound environment or host–biomaterial interface. This restoration would allow for more expedited wound closure without excessive fibrosis at the wound or implant site.

Currently, there is a dearth of literature covering the effects of mitochondrial therapies in other key support cells of granulation tissue development, such as vascular endothelial cells or epidermal cells [181]. Investigations into these cell types could elucidate other benefits that introducing healthy exogenous mitochondria could yield in promoting a regenerative granulation tissue environment.

### Potential for mitochondria in avoiding chronic inflammation

Macrophages play a variety of vital roles in inflammation and can adopt a broad spectrum of activation states depending on their environment. There is a substantial interest in promoting the polarization of pro-inflammatory macrophages (M1) to anti-inflammatory, reparative macrophages (M2) to modulate chronic inflammatory diseases or to promote native tissue integration in biomaterial implantation. When macrophages polarize to M1, their mitochondrial function is disrupted, producing high levels of ROS to kill bacteria. However, in mouse and human models, macrophages were activated with lipopolysaccharides and interferon-γ to induce macrophage polarization to an inflammatory state. These macrophages showed a significant reduction in their mitochondrial function and failed to restore into M2 even in the presence of interleukin-4, a cytokine that induces macrophages to polarize into M2. Inhibiting the production of ROS and improving mitochondrial respiratory function increased the ability of macrophages to repolarize to M2 [182].

As previously discussed, a study involving foam cell macrophages indicated that mitochondrial therapies could restore the ability of macrophages to repolarize into the M2 phenotype. Foam cells undergoing lipid accumulation *in vitro* were found to have diminished phagocytic capabilities and pro-inflammatory release profiles. When treated with isolated exogenous mitochondria, pro-inflammatory M1 markers TNF and NLRP3 were reduced, and M2 markers arginine and IL-10 were restored [183].

These findings were further corroborated in the context of neuroinflammation. An aforementioned sepsis model found that inflammation-induced mitochondrial dysfunction in microglia polarizes them to M1 phenotype. The introduction of exogenous mitochondria restored mitochondrial function and allowed the microglia to repolarize to restorative M2 phenotype [167].

Dextran-TPP-coated mitochondria reduced the pro-inflammatory potential of activated M1 macrophages *in vitro*, reducing pro-inflammatory cytokine release and promoting M2 phenotype shifts. Additionally, in a mouse atherosclerosis model, dextran-TPP-treated mitochondria were shown to be co-localized with macrophages within atherosclerotic plaques, reducing lesions, improving lipid profiles and reducing end-stage liver damage [184].

Thus, mitochondrial dysfunction within immune cells can prevent the body’s natural shut-off switch for inflammatory responses. By allowing the predominant cell type responsible for chronic inflammation (macrophage or macrophage-like cells such as microglia in the brain) to shift from pro-inflammatory effector and signaling cells to remodeling and anti-inflammatory cells, inflammation can be further resolved, preventing the development of chronic inflammation.

### Potential for mitochondria in the avoidance of foreign body response and fibrosis

The host–biomaterial continuum is a sequence of overlapping and interconnected events. The early immune responses to the biomaterial dictate the levels of success at the granulation tissue development phase and, ultimately, if fibrosis will occur. Upon poor resolution of acute inflammation, chronic inflammation will occur, preventing proper regeneration of native tissue. When inflammatory signaling does not transition from attack signaling to restorative signaling, the foreign body response and, ultimately, fibrosis will occur, preventing integration with the host and potentially leading to device failure. The foreign body response to a biomaterial entails macrophages engaging in a process named ‘frustrated phagocytosis’. Upon failure to engulf the biomaterial, macrophages adhere to the surface and fuse to one another, forming foreign body giant cells [8]. This process results in continued inflammatory signaling, poor support cell infiltration to the host–biomaterial interface and continued oxidative and enzymatic attack toward the implant. Mitochondrial transplantation offers a potential new method for improving the outcome of device integration into the host by modulating the body's response to an implanted biomaterial. Mitochondrial transplantation is still in the early stages of investigation, with the main thrust of research being toward cardioprotection post-infarction. However, the ability of mitochondrial transplantation to lower immune responses through a myriad of avenues presents tremendous potential for new therapeutic techniques in lowering the adverse reactions to implants and promoting wound healing. Thus, it is critical that more research be done on the effect of mitochondrial transplantation on the overall reduction in immune response upon biomaterial implantation and the promotion of wound healing at the implant microenvironment.

## Unresolved questions and obstacles regarding mitochondrial therapies

Mitochondrial transplantation has been reported to increase cell viability and native tissue efficiency and restore oxidative damage in numerous studies and a wide array of tissue types. Despite the widely reported benefits positing that mitochondrial transplantation is a ‘miracle cure’, the actual mechanisms of these benefits are still relatively unclear. Additionally, the best methods of sourcing, preparation and optimal mode of delivery of exogenous mitochondria have yet to be determined, much less standardized. While the current results and potential future applications are groundbreaking and exciting, it would be remiss not to discuss the drawbacks, potential failure points and current limitations of mitochondrial transplantation. Several major gaps remain in our understanding of mitochondrial transplantation, as well as obstacles yet to be overcome. These will be discussed in detail in the following paragraphs.

### Calcium ion sensitivity of extracellular mitochondria

The first category of major questions that have yet to be fully answered is mechanistic in nature. First, how do the transplanted mitochondria survive in an extracellular environment long enough to be taken in by host cells? Mitochondria are highly sensitive to ionic concentrations due to their high membrane potential, and calcium ions, in particular, are highly regulated by uniporter mechanics (mitochondrial calcium uniporter). This high electrical gradient, maintained around 180 mV, is critical to the mitochondria’s ability to generate ATP via pumping protons across the ion-permeable inner membrane [185]. Mitochondria are so sensitive to calcium ion concentrations that even within the micromolar Ca^2+^ range, calcium ions can build up within the mitochondrion, causing heightened permeability in a phenomenon dubbed the ‘permeability transition’, which can result in the opening of non-selective, large pores. This permeability transition, if unmitigated, causes massive swelling of the mitochondrial membranes. Due to the smaller surface area of the outer membrane in relation to the inner membrane, this swelling first causes destruction of the outer membrane before the inner membrane. It then eventually leads to a great decrease in membrane potential and the washing out of NAD/NADH, fully destroying the respiratory capability of the mitochondria. Even when polysaccharides and polypeptides within the extracellular space can mitigate mitochondrial swelling, mitochondrial pore opening can cause irreversible disruption to their oxidative capabilities [186]. The Ca^2+^ concentration characteristic of blood is ∼1.8 mM, a high enough concentration that failsafe mechanisms within mitochondria can no longer prevent permeability transition [187]. In 2015, a study investigated the viability of isolated mitochondria after exposure to the ionic concentrations found in the blood. The only instance in which mitochondrial viability was preserved was when the mitochondrial calcium transporters were blocked [188, 189]. This line of questioning establishes a ticking clock wherein mitochondria must provide the reported benefits before rapidly succumbing to the high physiological levels of Ca^2+^ within the extracellular space.

### Mitochondrial uptake mechanisms and number within target cells

**Figure 4.**
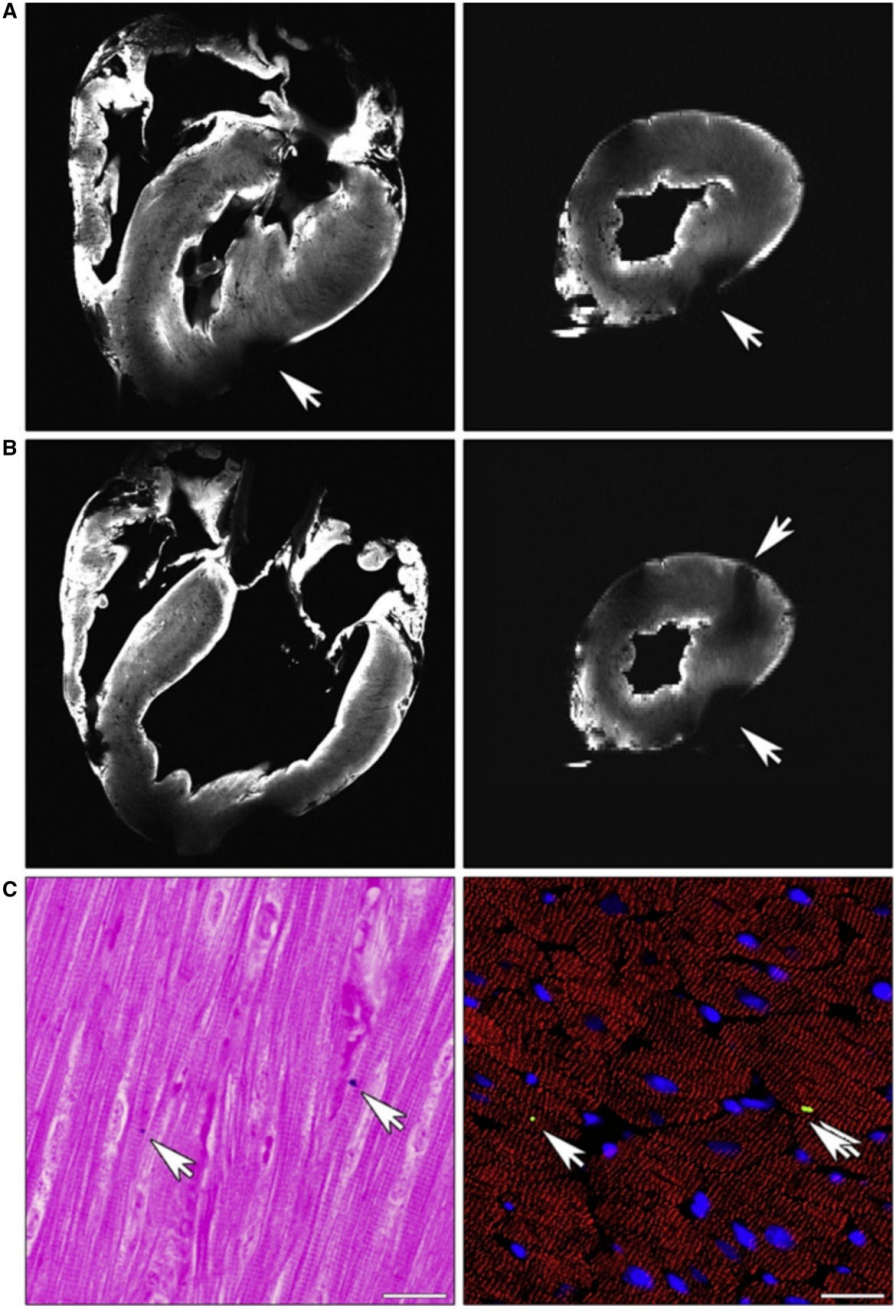
Limited mitochondrial uptake into heart cells after 4 weeks post-injection. Reprinted from Kaza *et al.* [192], Copyright (2017), with permission from Elsevier.

Even if isolated mitochondria can survive the Ca^2+^ levels in the bloodstream, penetrate the endothelial layer and enter the target cells, it is still unclear how so few of the remaining mitochondria actually provide the reported benefits. The reported percentages of mitochondria found within the cell compared to the total injected range between 3% and 7% based on several separate reports from various labs [79, 83, 190]. In the human heart, the turnover rate for the total amount of ATP is <1 min. Providing any nominal benefit to a cardiac myocyte would require thousands of viable mitochondria producing ATP at a highly efficient level [191]. Immunostaining of a representative tissue section in a pig study showed only three human mitochondria after 4 weeks post-injections in an entire visual field of myocytes (Fig. 4) [192].

**Figure 5.**
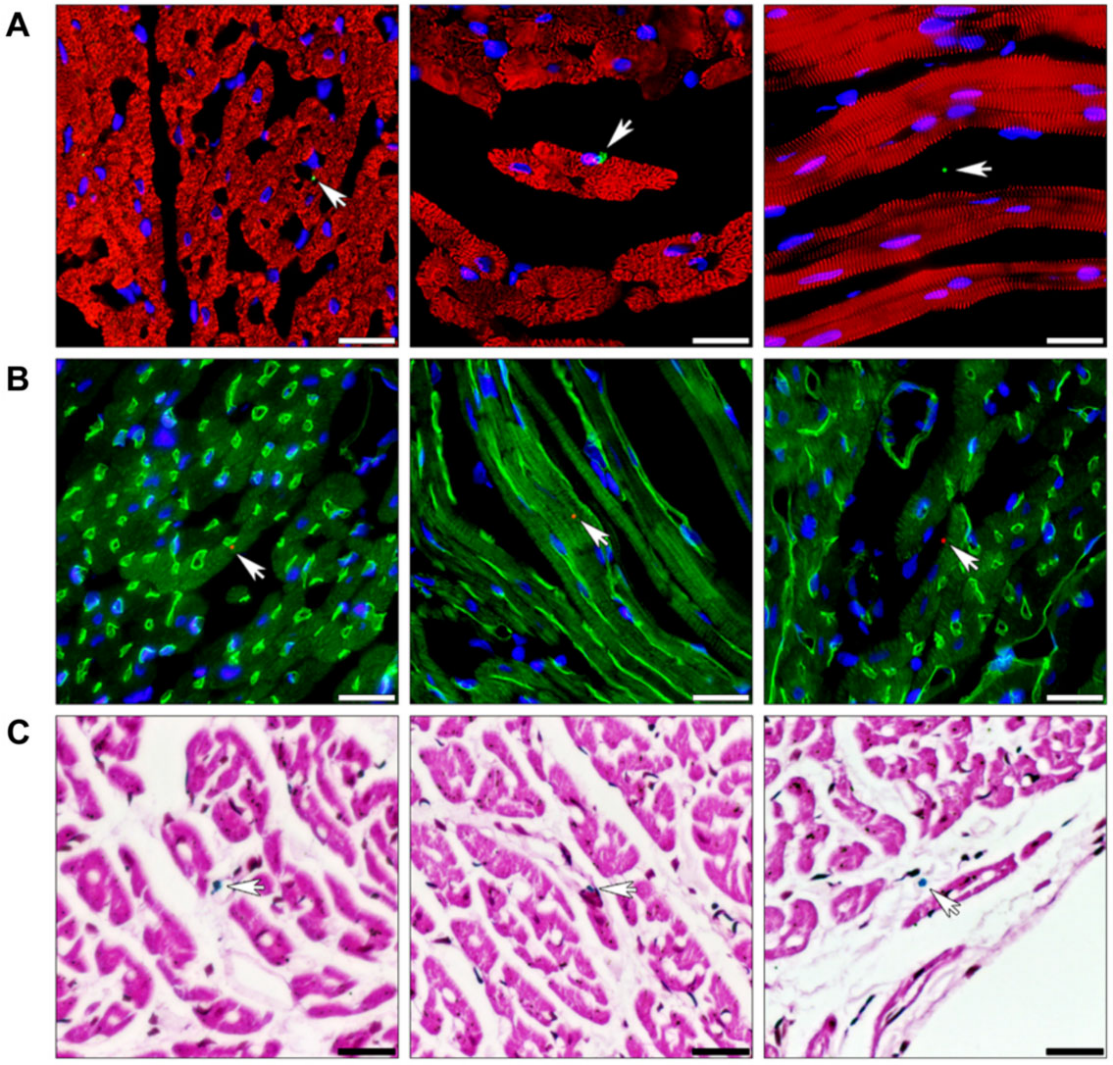
Insufficient mitochondrial uptake into host cells to explain metabolic rescue to tissue. Adapted with permission from Cowan *et al.* [84].

Additionally, fluorescence microscopy conducted after coronary perfusion with human mitochondria in a rabbit heart model showed a wide dispersion of mitochondria sparsely distributed along the vasculature and capillary interstitial spaces. In this study, few mitochondria were spotted adjacent to cardiac myocytes, but a still insufficient level to significantly increase ATP production (Fig. 5) [84].

In both earlier and other cases, there is insufficient evidence to suggest that enough mitochondria are entering the target cells to provide the reported benefits through the mechanisms currently proposed (ATP production/metabolic repair). Interestingly, while there is low intake into target cells after mitochondrial transplantation, several key inflammation markers are decreased, as mentioned previously, potentially hinting at the lowering of immunopathology as an explanation for the observed benefits, as opposed (or supplementary) to the current ATP-metabolism rescue-based explanations [83, 93, 193].

### Sourcing and storage of mitochondria for clinical use

Another major obstacle in mitochondrial transplantation is the matter of sourcing and storage of isolated mitochondria. Quality control parameters include low Ca^2+^ concentrations in the media, maintaining a temperature below 4°C and preserving mitochondria outer membrane potential [165]. Storage on ice for more than an hour results in damaged membrane viability and, thus, a loss of capability to generate ATP in isolated mitochondria [194]. Additionally, mitochondria with compromised membranes will not provide benefits nor be taken in by host cells, and free-floating mtDNA can trigger an immune reaction. Including cytosolic components within the mitochondrial solution is another trigger for an immune attack [165]. Most clinical approaches use autologous sourcing, wherein a biopsy of the patient's muscle tissue is used as the mitochondria source. Typically, the mitochondria are isolated as the patient is on the operating table, requiring fast isolation of the mitochondria and limiting the ability to run quality control before transplant. These obstacles show the need for an alternate sourcing solution or an ability to collect, store and test mitochondria before surgery. A potential sourcing solution could be collecting proliferating primary cells (such as fibroblasts), expanding cell culture, isolating the mitochondria from the cell population for transplant and testing viability and purity before surgery. Long-term storage solutions involving dimethyl sulfoxide (DMSO), glycerol or trehalose have also been investigated, showing some promise in preserving oxidative phosphorylation. However, cytochrome *c* retention was reduced upon thawing, which could lead to an induction of inflammation upon implantation [194]. These cold-storage techniques show promise, but it has yet to be determined whether mitochondria will be able to be stored with these methods and still provide a clinical benefit while not inducing an immune response, either from cytochrome *c* release, mtDNA release or the freezing agent included in the solution. Both DMSO and glycerol have been found to be cytotoxic at sufficient concentrations, and the cold-storage techniques each utilize concentrations that meet or exceed the cytotoxic limit.

There is currently an extensive and growing body of knowledge on the topic of mitochondrial transplantation. However, the above questions and obstacles provide opportunities to advance scientific understanding. Developing our understanding and optimizing techniques will bring mitochondrial transplantation from theory and closer to practical utilization in the clinic to alleviate tissue damage and possibly help promote host tissue integration with implanted biomaterials.

## Proposed alternate mechanisms and approaches

The following paragraphs will explore possible alternate mechanisms that may explain why investigations into mitochondrial transplantation yield such promising results. These potential other routes of mediating tissue damage elucidate avenues of inquiry that could lead to new therapies and new technology to facilitate even more efficacy from mitochondrial transplantation. As mentioned, these mechanisms could work synergistically with the previously proposed mechanisms of ATP replenishment. For example, mitochondrial transplantation could have an immunoprotective effect, shielding the damaged tissue from excessive inflammation and allowing the affected cells to regenerate their ATP-producing capabilities without further oxidative damage from host immune cells.

### Neutrophil-focused mitochondrial therapies

The full extent of neutrophil involvement in immunopathology and host–biomaterial rejection is, to this day, not fully understood and has been a hot topic of investigation for the past few years. They have been shown to not merely be effector cells, recognizing foreign materials and killing them, but coordinators of the initial immune response, signaling more neutrophils to perform varied and specialized roles or signaling for other immune or support cells to begin later phases of the healing continuum [43]. Previous studies on mitochondrial transplantation have hinted at an effect on neutrophil involvement in the microenvironment. Still, since the focus of investigation has been primarily on the target parenchymal cells, no further in-depth study into neutrophil dynamics has been performed. A study investigating cold ischemia time in murine heart transplantation showed significantly less neutrophil infiltration into tissues upon injection of isolated mitochondria [195]. Neutrophil infiltration was used as a metric to measure necrosis; however, the question remains: could this be a causative relationship? Instead of there being less neutrophil involvement because there is less necrosis, could the inverse be true? Previous studies have also shown significant decreases in inflammatory markers such as IL-6, IL-10, TNFα, MCP-1 and hsCRP compared to injured tissue that did not receive mitochondrial transplantation [83]. These findings of lowered inflammation markers have been corroborated in many other studies on various other tissues, such as the post-ischemia–reperfusion lumbar spinal cord, wherein a study also concluded that IL-6 and TNFα levels were reduced after mitochondrial transplantation [196]. Other papers reported that no inflammatory markers were upregulated compared to the controls to emphasize the lack of immune attack of exogenous mitochondria. One such paper wherein bronchoalveolar lavage (BAL) was assayed for cytokine upregulation post-mitochondrial transplantation notably lists under their limitations: ‘An additional limitation is that BAL was collected for cytokine analysis at 24 h of reperfusion. Cytokine release peaks at the early reperfusion; therefore, cytokine upregulation in our study may be decreased’ [197]. Whether or not it is purely from lower levels of necrosis due to mitochondrial transplantation, there is an evident amelioration of post-injury inflammation that warrants further investigation. It is important to note that in nearly all reports of lower inflammatory cytokine expression levels after mitochondrial transplantation, IL-6 is knocked down. IL-6 is of particular significance because it is considered the primary stimulator of most acute phase proteins, drastically affecting the initial acute inflammatory response [198]. IL-6 has been implicated in neutrophil recruitment and trafficking toward inflammation sites. It has also been shown to prime the immune response away from resolution and towards chronic inflammation [199–201]. In severe pathological inflammation, IL-6 is mainly produced by neutrophils and other myeloid leukocytes, signaling a dire prognosis in many health conditions such as severe COVID-19 [202, 203].

Thus, the knockdown of IL-6 and other key acute inflammatory cytokine levels due to mitochondrial transplantation may have promising implications for wound healing and tissue–device dynamics. By lowering the initial immune attack to the injury microenvironment, mitochondrial transplantation may mediate tissue damage from an immunoprotective mechanism and not just by supplying additional ATP production.

### Mitochondrial therapeutics for targeting macrophage polarization

Another potential avenue for mitochondrial transplantation involves a focus on macrophages and monocytes. Mitochondrial dysfunction and oxidative stress have been demonstrated to prevent macrophages from polarizing back into their anti-inflammatory M2 phenotype after pro-inflammatory stimulus [182]. It could be that macrophages within an environment of chronic inflammation and ensuing oxidative stress accumulate damage to their respiratory pathways, causing them to remain in an inflammatory phenotype, thus creating a vicious cycle of chronic inflammation [204]. A recent study investigating the effects of mitochondrial transplantation on macrophage lipid accumulation with the aim of preventing foam cell development found promising results in enhancing phagocytic capacity and anti-inflammatory marker expression. The study indicated anti-inflammatory markers expressed by M2 macrophages (or M1 macrophages transitioning to M2), such as IL-10 and arginine [183].

Additionally, NLRP3 and TNF-α were shown to be decreased in mitochondria-treated macrophages undergoing lipid overload. However, it is important to note that control macrophages increased pro-inflammatory markers when given exogenous mitochondria and that the anti-inflammatory benefit seems to only occur on macrophages already primed for the M1 phenotype [183]. This study is in line with a previous study in which mitochondrial transfer from MSCs into monocyte-derived macrophages enhanced phagocytic capabilities and shifted macrophage polarity to M2 [80]. These findings suggest that mitochondria are not simply power plants to boost cell respiration but can shift immune cell phenotypes from pro-inflammatory to anti-inflammatory. Utilizing this potential could put a halt to pro-inflammatory feedback loops that doom implant–tissue interfaces and injuries to chronic inflammation and, inevitably, fibrosis.

### Mitochondria as an oxidative therapy

Another fairly ubiquitous finding in mitochondrial transplantation research is a reduction in ROS at the injury site. A large body of evidence suggests that mitochondria can not only scavenge endogenous ROS produced from their own respiratory chain but can also scavenge ROS from other sources using the same systems. This phenomenon even extends outside the cell, with experimental evidence indicating that mitochondrial antioxidant systems can scavenge superoxide radicals and hydrogen peroxide from the surrounding microenvironment. For example, brain mitochondria were found to be able to remove exogenously added H_2_O_2_ at a high rate of around 6.5 nmol/min/mg protein. These findings suggest that mitochondria can scavenge ROS at a rate nearly 3–12 times higher than what is produced by mitochondria under physiological conditions [205, 206]. This phenomenon has yet to be further investigated as a potential therapeutic. However, the evidence presented by these studies demonstrates the ability of mitochondria to act as a biological antioxidant, scavenging ROS and protecting tissues from oxidative stress. It could very well be that transplanted mitochondria, even if they are not taken into the target cells or support cells, act as an antioxidant, thus explaining the reduced tissue damage and attenuation of inflammation.

## Engineering approaches to mitochondrial therapeutics

In addition to opportunities for development in biological understanding, optimizing mitochondrial transplantation also provides engineering challenges. Currently, mitochondrial transplantation has primarily been performed via intravenous injection for *in vivo* applications. This method results in a large spread and sparse distribution of mitochondria within the target tissue, with reports stating that the total mitochondrial intake into target cells is between 3% and 7% [79, 83, 190]. Additionally, the high Ca^2+^ concentrations present in the extracellular spaces that the mitochondria must pass through present another potential route to explore for engineering solutions. *In vitro* studies, especially those in which the cellular media is Ca^2+^ free or has low Ca^2+^ concentrations, show a larger intake of mitochondria into host cells and higher mitochondrial viability overall. If a material could be engineered to both keep transplanted mitochondria within the target environment and protect the mitochondria from Ca^2+^, the efficacy of mitochondria transplantation therapies could potentially be increased tremendously. A scaffold or semi-permeable patch that could be grafted onto the injured area, allowing mitochondria to flow to the affected area without being carried away by blood flow, would likely allow for better host intake. Particularly, a material capable of limiting the influx of Ca^2+^ into the area would help preserve mitochondrial viability. The potential for mitochondrial transplantation is thus not limited to regenerative medicine, as there is tremendous potential for tissue engineering and biomaterial approaches to help optimize these strategies for the clinical and operating rooms.

## Conclusion

Mitochondrial transplantation is a growing field of study with great potential. This burgeoning therapy has shown promise in a vast array of areas, such as treating ischemia–reperfusion damage in a wide range of tissues, nerve damage via crushing and even depression and neurological conditions. However, there are still several critical questions that must be addressed, either mechanistic or practical in nature. Additionally, there is currently unrealized potential in the field of engineering to find solutions to the present obstacles limiting the efficacy of mitochondrial transplantation. Through interdisciplinary efforts and creative problem solving, a new frontier of scientific and technological development can be ushered in. This new frontier has the potential to bring mitochondrial therapies closer to becoming viable clinical treatments that could change the course of wound healing and host–biomaterial interactions from mere repair towards complete host tissue regeneration. This change would allow for better patient clinical outcomes by lowering scar tissue formation and allowing fuller tissue–device integration.

*Conflicts of interest statement*. No conflicts of interest present from the authors.
